# Serum catalase, thiol and myeloperoxidase levels in children passively exposed to cigarette smoke

**DOI:** 10.1186/s13052-019-0652-8

**Published:** 2019-05-09

**Authors:** Emel Torun, Feyza Ustabas Kahraman, Ahmet Zaid Goksu, Aysel Vahapoglu, Zeynep Ebru Cakin

**Affiliations:** Bezmialem Vakif University Medical Faculty, Departments of Pediatrics, Istanbul, Turkey

**Keywords:** Passive smoking, Catalase, Thiol, Myeloperoxidase, Children

## Abstract

**Background:**

Free radicals found in cigarette smoke can harm all tissues and cellular structures in the human body. Passive smoking increases free radical production, leads to the depletion of antioxidants and increases oxidative stress which causes lipid peroxidation. Many studies have been conducted to determine the effects of passive smoking on antioxidant enzymes and lipid levels in adults, but pediatric studies on this topic are few. In our study, we compared the levels of antioxidants, oxidants, total and LDL cholesterol in children exposed to passive cigarette smoking with a healthy control group that was not exposed to passive smoking.

**Methods:**

A total of 41 children (4–17 years of age, 24 girls and 17 boys) exposed to passive smoking and 18 healthy girls and 12 healthy boys were included in this study. Secondhand smoking was confirmed via measurement of the cotinine/creatinine ratio. Various sociodemographic characteristics of patients were recorded. The levels of catalase, thiol, myeloperoxidase were measured to determine the antioxidant and oxidant levels in children, while the levels of total cholesterol and LDL cholesterol were measured to determine the alterations in lipid profile.

**Results:**

The groups were similar in regard to demographic characteristics. Myeloperoxidase levels were significantly higher in the passive cigarette smoking group compared to the non-exposure group; however, catalase and thiol levels were similar. In regard to lipid profile, the levels of total cholesterol and LDL cholesterol were also similar in those with and without exposure to passive smoking.

**Conclusions:**

Our findings suggest that the effects of passive smoking initially influence oxidants (MPO), but not antioxidants (thiol and catalase). However, it is apparent that passive smoking adversely affects oxidative balance in children and this may lead to the development of various diseases which could cause significant morbidity and mortality.

## Background

Environmental tobacco smoking is defined as passive exposure to smoke released from a burning tobacco product. Passive smoking (also termed as secondhand smoke) is harmful to human health at all stages of life, especially in childhood. Studies have shown that 43% of children have at least one smoker in their household [1]. Seven hundred million children worldwide are passively exposed to the harmful effects of smoking [2]. Passive cigarette smoking is associated with developmental disorders, cardiovascular diseases, pulmonary diseases, stroke and carcinogenesis. Other adverse effects include premature birth, perinatal mortality, fetal growth retardation, and increased frequency of sudden infant death syndrome [3, 4].

Both the gases and the tar particles in tobacco smoke act as oxidants and prooxidants, and induce the production of high levels of reactive oxygen species (ROS), reactive nitrogen species (RNS), and free radicals; all of which are known to cause cellular damage and exert toxic effects to tissues and organs [5]. Free radicals are constantly produced in the body and they are normally rapidly neutralized by antioxidants, resulting in an equilibrium called oxidative balance. An increase in free radicals disrupts this balance in favor of oxidant formation, leading to increased oxidative stress, which has been shown to play an important role in passive smoking-related conditions and has also been implicated in the development of cancer, autoimmune diseases, rheumatoid arthritis, and cataract formation, as well as cardiovascular and neurodegenerative diseases [6, 7].

The primary antioxidant enzyme, CAT (Catalase), is found especially in erythrocytes but also in hepatocytes and the kidneys. Catalase converts H_2_O_2_ to H_2_O in peroxisomes and is one of the most reactive enzymes in the human body; converting around 6 million H_2_O_2_molecules to H_2_O per minute [8]. This reaction is critical, as failure to destroy H_2_O_2_ results in the formation of OH free radicals via the Fenton reaction [9], leading to DNA damage and lipid peroxidation [10]. Thiols are sulfhydryl-containing organic compounds that serve as key components of intracellular and membrane antioxidant systems. Thiols have a number of mechanisms in which they exhibit their antioxidant activity, including thiol/disulfide reactions, metal chelation and their involvement in glutathione reactions as substrates. They comprise the largest group of plasma antioxidants in vivo*,* and therefore, are critical to oxidative balance [11]. The enzyme myeloperoxidase (MPO) is an iron-containing hemoprotein carried by neutrophils. MPO shows its effects at sites of inflammatory tissue damage and is a critical component of the inflammatory response through its role in a process called ‘oxidative burst’ which produces ROS to destroy pathogens [12].

Various studies have shown that cigarette smoking is associated with lipid peroxidation [13], increased oxidative stress and inflammation [14, 15]. Furthermore, cigarette smoking has been shown to inhibit CAT activity [16], reduce thiol levels [17], and increase MPO activity [18]. However, there are only a few studies in which the effects of passive smoking on the oxidative balance and lipid levels of pediatric patients were assessed. In this study, we aimed to determine the effects of passive smoking on lipids and parameters associated with oxidative stress (catalase, thiol and MPO levels) in healthy children.

## Methods

This study was conducted between January 2015 and January 2016 at the Bezmialem Vakıf University Medical Faculty Hospital Child Health and Diseases Polyclinic, Istanbul, Turkey. Ethical approval was obtained from the Local Ethics Committee of the University (decision no: 42/15, date: 25.09.2013). Written informed consent was obtained from the families of all children included in the study.

Our cross-sectional study enrolled 24 girls and 17 boys, between the ages of 4 and 17 years, who had been exposed to tobacco smoke (at least five cigarettes per day) during the last 6 weeks before inclusion into the study. Exposure to passive smoking was confirmed based on the cotinine/creatinine ratio from urine obtained in the first morning that patients were included into the study. Children with infections, chronic diseases, family history of hereditary lipidemia, drug use, tobacco or alcohol consumption, and those receiving parenteral nutrition were excluded as these conditions may affect lipid levels and oxidative balance.

The control group consisted of 12 boys and 18 girls within the same age range but not exposed to passive cigarette smoke for at least 6 weeks prior to inclusion into the study. Non-exposure was confirmed based on the cotinine/creatinine ratio from the first morning void urine sample.

The demographic characteristics, socioeconomic status, and smoking habits of the families of the participants were assessed in both groups. Serum and urine samples were collected for biochemical tests. The body mass index (BMI, kg/m^2^) of all participants was calculated.

### Measurement of cotinine and creatinine in urine samples

The first morning void urine samples of all participants were frozen at − 20 °C until measurement. The urine cotinine level was measured using an enzyme-based immunoassay (Thermo-Fisher Scientific, Waltham, MA, USA), and the urine creatinine concentration was measured according to a calorimetric method. Passive smoking exposure was assessed by calculating the urine cotinine/creatinine ratio rather than the absolute value of either compound, due to the high variability in urine volume among the participants. There is no cut off value for cotinine/creatinine ratio. We compared cotinine/creatinine mean between smoke exposed group and the control group.

### Biochemical tests

Total and low-density lipoprotein (LDL) cholesterol levels were measured using a homogeneous colorimetric enzyme technique (Cobas 800; Roche, Basel, Switzerland).

#### Measurement of CAT activity

CAT activity, as an indicator of antioxidative status, was measured based on the degradation of H_2_O_2_ via the method developed by Aebi et al. [26]. CAT levels were quantified spectrophotometrically on a Siemens ADVIA 1200 spectrophotometer and the results were expressed as IU/mL**.**

#### Measurement of thiol levels

Free sulfhydryl groups (−SH) in serum were measured using a modification of the method of Hu et al. [19] and quantitated using an autoanalyzer (ADVIA 1200; Siemens) according to a method reported previously [20]. The results were expressed as μmol/L.

#### Measurement of MPO activity

An autoanalyzer (ADVIA 1200; Siemens) was used to measure MPO activity, according to the method reported by Krawisz et al. [21]. The results were expressed as IU/mL.

### Statistical analysis

The NCSS 2007 software (NCSS Statistical Software, Kaysville, UT, USA) was used for all statistical analyses. Data obtained by descriptive statistical methods (mean, standard deviation, median, frequency, ratio, minimum, maximum) which showed a normal distribution were evaluated using the Student’s t test in two-group comparisons. Data with a non-normal distribution were compared using the Mann Whitney U test for two-group comparisons. Pearson’s chi-square test and Yates’ continuity correction test (Yates corrected chi-square) were used to compare qualitative data. Intervariable correlations were evaluated using Spearman’s correlation analysis. *P* values of < 0.05 were considered to indicate statistical significance.

## Results

The study included a total of 29 boys (40.8%) and 42 girls (59.2%) aged between 4 to 17 years (mean ± SD = 10.17 ± 3.61 years). The BMI values of participants ranged from 12.89 to 28.40, with a mean of 17.92 ± 3.42 (Table 1).

**Table 1:** Demographic characteristics of all children included in the study

		Min–max	Mean ± SD
Age (years)		4–17	10.17 ± 3.61
BMI (kg/m^2^)		12.89–28.40	17.92 ± 3.42
		n	%
Sex	Male	29	40.8
	Female	42	59.2
	< 1000 TL	20	28.2
Family income (TL)	1000–2000 TL	37	52.1
	> 2000 TL	14	19.7

*TL* Turkish liras

In the study group, 23 children (47.9%) were exposed to passive smoking via the father, 12 (25%) via the mother, and 13 (27.1%) via other persons living in the household. Twelve of the patients (29.3%) were exposed to 5–10 cigarettes per day, while 29 (70.7%) were exposed to 10–20 cigarettes per day.

Age, gender, BMI, and family income showed no statistically significant differences between the study and control groups (*p* > 0.05). Only the urine cotinine/creatinine ratio was significantly higher in the study group (*p* < 0.01). In terms of lipid parameters, the levels of total cholesterol and LDL cholesterol were similar in both groups (Table 2).

**Table 2:** Comparison of the study and control groups in terms of demographics and laboratory findings

		Study Group (*n* = 41)	Control Group (*n* = 30)	***P***
Age (years)	*Mean ± SD*	10,15 ± 3,77	10,20 ± 3,45	^*a*^ *0,951*
	*Min-Max (Median)*	5–17 (10)	4–16 (10,5)	
BMI (kg/m^2^)	*Mean ± SD*	18,07 ± 3,64	17,73 ± 3,19	^*b*^ *0,694*
	*Min-Max (Median)*	12,89–28,4 (16,62)	13,13–24,28 (16,43)	
Total Cholesterol (mg/dl)	*Mean ± SD*	149,38 ± 24,25	148,50 ± 24,9	^*a*^ *0,885*
	*Min-Max (Median)*	96–206 (149,5)	90–207 (149)	
LDL Cholesterol (mg/dl)	*Mean ± SD*	89,32 ± 23,91	83,59 ± 21,9	^*a*^ *0,322*
	*Min-Max (Median)*	43–150 (85)	32–126 (89)	
Cotinine/creatinine ratio (ng/mg)	*Mean ± SD*	1,27 ± 1,16	0,22 ± 0,66	^*b*^ *0,001*
	*Min-Max (Median)*	0–5,19 (0,78)	0–2,52 (0)	
		n (%)	n (%)	
Gender	Male	17 (41,5)	12 (40,0)	^*c*^ *1,000*
	Female	24 (58,5)	18 (60,0)	
	< 1000 TL	13 (31,7)	7 (23,3)	^*d*^ *0,726*
Income	1000–2000 TL	20 (48,8)	17 (56,7)	
	> 2000 TL	8 (19,5)	6 (20,0)	

^a^Students t-test

^b^Mann Whitney U Test

^c^Yates Continuity Correction Test

^d^Pearson Chi-Square Test

*TL* Turkish liras

When the levels of oxidative stress parameters were compared, only MPO activity was found to be significantly higher in the study group compared to controls (*p* = 0.039) (Table 3).

**Table 3:** Comparison of thiol, CAT, and MPO levels in regard to groups

		Study group (n = 41)	Control group (n = 30)	***P***
Thiol (umol/L)	*Mean ± SD*	0.63 ± 0.56	0.51 ± 0.19	^*a*^ *0.629*
	*Min-Max (Median)*	0.29–2.88 (0.40)	0.28–1.00 (0.43)	
CAT (IU/mL)	*Mean ± SD*	51.68 ± 57.14	40.69 ± 41.63	^*a*^ *0.926*
	*Min-Max (Median)*	4.82–189.17 (17.46)	6.55–140.28 (20.51)	
MPO (IU/mL)	*Mean ± SD*	370.30 ± 449.04	165.39 ± 165.89	^*a*^ *0.039*
	*Min-Max (Median)*	50.81–2268.61 (173.98)	23.86–587.07 (93.12)	

^*a*^ *Mann Whitney U test*

## Discussion

In the current study, we evaluated the effects of passive smoking on cholesterol levels and several oxidative stress parameters of pediatric patients. Our groups were similar at baseline in terms of age, sex, socioeconomic characteristics and BMI. Comparison of groups indicated that, while MPO activity was increased in those with passive exposure to cigarette smoke, the levels of total cholesterol, LDL cholesterol, thiol and CAT were similar among groups.

Diseases caused by smoking or exposure to environmental cigarette smoke have become a serious public health problem in adults and children. In a study published by the World Health Organization (WHO) which was conducted in 192 countries, it was reported that 40% of children, 35% of women, and 33% of men are exposed to passive smoking, predominantly in the home and work environments [22]. As we only included pediatric patients in the current study, we evaluated the exposure of children to secondhand smoke in the home environment, and the changes in thiol, CAT and MPO levels were utilized as measures of antioxidant and oxidant levels. We also compared the differences between these parameters in children who were exposed to lower and higher levels of smoke; however, no differences were found regarding the parameters measured.

When the body is exposed to oxidative stress, it responds by activating its antioxidant defense systems, including CAT which is a member of the primary antioxidant system. The levels of several plasma proteins, including albumin, as well as those of antioxidants such as uric acid, bilirubin, and ascorbic acid are also elevated by oxidative stress [23]. These molecules act in concert to target the chronic inflammation caused by prolonged exposure to cigarette smoke. For example, increased CAT levels have been demonstrated in chronic smokers [24]. However, Zhou et al. found that compared to non-smokers, the levels of superoxide dismutase, glutathione peroxidase and CAT, together comprising the main plasma antioxidant system, were lower in smokers, most probably due to being overwhelmed by excessive free radical production [25]. Similarly, in another study, although the glutathione peroxidase levels of smokers were similar to controls, superoxide dismutase and CAT were found to be lower in individuals exposed to cigarette smoke. The authors explained the reduction in the levels of antioxidants with extreme oxidative stress possibly causing the breakdown of antioxidants in active smokers [26].

Based on current knowledge, thiols are considered as the most potent plasma antioxidants in vivo due to their sheer amount measured in body fluids. Studies on the plasma levels of thiol antioxidants in adult patients exposed to active and passive smoking have reported high levels of homocysteine, and thus a risk for atherosclerosis, in both groups [27]. A study in which the levels of glutathione and its precursor amino acid cysteine were compared between former and active smokers, high levels of both compounds were measured in former smokers [28]. A reduction in the number of cigarettes smoked was found to have no effect on antioxidant system components in smokers; but when smoking was discontinued, oxidative stress was decreased and antioxidant enzyme levels were increased. This finding shows that even low exposure to smoke has significant effects on oxidative balance.

The literature on this topic demonstrates that oxidative stress is remarkably increased among smokers [29–31]. Furthermore, children exposed to even low amounts of smoke have been shown to have increased oxidative stress in a study comprised of children aged 4 to 6 years [32]. In the current study, thiol and CAT levels, as measures of antioxidant capacity, were similar in those with and without exposure to secondhand smoke, while MPO activity was found to be increased in children exposed to smoke in their home environment. Although various studies have suggested that both oxidant and antioxidant levels are changed among those subject to cigarette smoke [33–35], there are several studies that have reported findings similar to ours. Yıldırım et al. reported a lack of difference in total antioxidant capacity between children (4–6 years) exposed to 5–50 cigarettes per day and those that were not exposed [32]. Kahraman et al. also observed a similar result in their study comprised of 80 children (40 exposed, 40 not exposed, aged 5–17 years). They found that total oxidant status and oxidative stress index were increased in children exposed to secondhand smoke; however, total antioxidant status was similar in both groups [36]. In the light of our results and the literature on this topic, it may be feasible to suggest that passive smoking initially effects oxidant levels, but not antioxidant levels. However, this does not necessarily mean that antioxidant capacity is unchanged in children exposed to smoke, it only shows that the balance between oxidants and antioxidants has changed in favor of oxidants. Considering that physiological oxidative balance is critical in tissue and organ function, it is safe to assume passive smokers have an underlying response to the oxidative imbalance caused by smoke, but the normal levels of antioxidants seen in these patients should be assessed with regard to the consumption needed to maintain oxidative balance. As such, a higher level or longer duration of exposure may be required in order to observe a significant reduction in antioxidant levels. While these assumptions may apply to the antioxidants in question, there are reports indicating that antioxidant vitamin levels are lowered in infants and children exposed to smoke [34, 37]. This reduction in the levels of micronutrients involved in antioxidant defense supports the conclusion that intrinsic antioxidant mechanisms respond to the increase in oxidative stress caused by passive smoking, while nutrient-based antioxidants (such as vitamins) may be decreased in response to increased oxidative stress.

It is well known that smoke inhalation causes lipid peroxidation through an increase in oxidative stress in either active or passive smokers [38, 39]. Smoking is also associated with higher levels of total cholesterol, LDL cholesterol and triglycerides, and lower levels of high- density lipoprotein (HDL) cholesterol [40]. In the current study, children subject to passive smoking had similar total and LDL-cholesterol levels. However, a systematic review conducted in 2011 showed that passive smoking may have significant effects on the lipid profile of children; even so, they did conclude that study designs limited evaluation in especially infants and those subject to maternal smoking [41]. Our findings are supported by several studies that reported similar lipid profiles in children (12–19 years old) exposed to secondhand smoke and those that were unexposed [42]. Furthermore, one particular study by Neufeld et al. suggested that the relationships shown between lipid profile and passive smoker status in children may be due to the absence of adjustments for confounding factors (such as diet and dyslipidemia). In their study, they found that only HDL cholesterol levels were increased in children (2–18 years old) exposed to environmental smoke, while other lipid parameters were not associated with smoking exposure [43]. Although we did not measure HDL cholesterol levels, the absence of a significant difference between our groups in terms of total and LDL cholesterol supports these results. However, the duration of exposure to secondhand smoke (in relation with child age and duration of smoker presence in the household) may be an important determinant on lipid profile in the long-term. Future studies would benefit from evaluating secondhand smoke in regard to the duration and frequency of the child’s exposure to the smoker(s) in the home environment.

When taken together, our results indicate that increased oxidative stress in children exposed to passive smoking may be balanced to some degree by an underlying increase in antioxidant capacity. However, the oxidant enzyme MPO was also found to be increased in the current study, which could lead to an increase in lipid peroxidation as MPO is known to be involved in lipid peroxidation, plaque formation and atherosclerosis in vessels [44]. Increased oxidative stress has significant effects on health as it is associated with the development of countless diseases, including atherosclerosis, diabetes, cancer and cerebrovascular diseases. Additionally, significant exposure to cigarette smoke in the childhood has been shown to be associated with higher risk for mortality due to chronic obstructive pulmonary disease [45]. Therefore, protecting children from exposure to cigarette smoke is crucial to prevent morbidity and mortality throughout their life.

There are several limitations in our study which must be discussed. Firstly, the number of participants are relatively low compared to more recent studies in this field. However, we only included new patients who had applied to our clinic, as inclusion of those who were already followed at our institution (and were informed about the dangers of passive smoking) was considered to be unethical. Secondly, there are various other parameters that are used for the evaluation of pro- and antioxidants, most of which were not measured in the current study. However, we measured thiol, CAT and MPO levels; which are considered to be among the most important parameters for determination of oxidative balance. Thirdly, exposure to secondhand smoke was defined as at least 5 cigarettes per day in the last 6 weeks; a definition which is solely based on parent reports and may not be accurate. However, there is no objective and ethical way to determine the level of exposure to secondhand smoke and to normalize distribution other than measuring the cotinine/creatinine ratio, which at least confirmed the presence of significant exposure. Lastly, although we excluded patients with active acute or chronic infections, the levels of MPO activity and other antioxidant levels may have been affected by other factors causing an increase in inflammation.

## Conclusion

In our study, the effects of passive smoking on the serum levels of oxidant and antioxidant enzymes in children and adolescents were evaluated based on comparison with an age-matched non-exposed group. In contrast to the many studies focused on the effects of passive smoking on the antioxidant system of adults, few such studies have been conducted in children and adolescents. The serum levels of the antioxidants thiol and CAT did not differ between the study and control groups, whereas MPO levels were higher in the former group. This shows that the oxidative balance of children exposed to passive smoking shifts towards the pro-oxidant state and suggests that oxidant levels are affected before alterations in antioxidants can be observed. Considering the previous studies in this field, it is apparent that passive exposure to cigarette smoke could cause the development of severe diseases in these children leading to increased morbidity and mortality. Our study provides further support for the implementation of precautions to prevent exposure to passive cigarette smoke in childhood.

## Abbreviations

BMI: Body mass index
CAT: Catalase
HDL: High- density lipoprotein
LDL: Low-density lipoprotein
MPO: Myeloperoxidase
RNS: Reactive nitrogen species
ROS: Reactive oxygen species
WHO: World Health Organization
